# Unlocking the Aromatic Profile of Wild-Grown Croatian Fennel: A Comparative Study of Essential Oils and Hydrolates

**DOI:** 10.3390/molecules31111867

**Published:** 2026-05-29

**Authors:** Ana Vučak, Ivana Generalić Mekinić, Petra Brzović, Danijela Skroza, Roberta Frleta Matas, Franko Burčul

**Affiliations:** 1 Department of Analytical and Environmental Chemistry, Faculty of Chemistry and Technology, University of Split, Ruđera Boškovića 35, 21000 Split, Croatia; 2 Department of Food Technology and Biotechnology, Faculty of Chemistry and Technology, University of Split, Ruđera Boškovića 35, 21000 Split, Croatia; gene@ktf-split.hr (I.G.M.); pbrzovic@ktf-split.hr (P.B.); danci@ktf-split.hr (D.S.); rfmatas@unist.hr (R.F.M.)

**Keywords:** *Foeniculum vulgare* Mill., plant parts, GC-MS, HS-SPME, antimicrobial activity, *trans*-anethole, *α*-phellandrene, fenchone

## Abstract

*Foeniculum vulgare* Mill. (fennel) is an edible and medicinal plant cultivated worldwide. Owing to its distinctive aroma and diverse biological activities, its essential oils (EOs) have been widely investigated. However, available data predominantly focus on cultivated fennel or commercial EOs, while comprehensive investigations of wild-growing Mediterranean populations—particularly comparisons among different plant parts—remain scarce. In this study, EOs obtained by hydrodistillation from stems, leaves, flowers, and fruits of native Croatian fennel, were chemically characterised using gas chromatography-mass spectrometry (GC-MS), while antimicrobial activity was evaluated using the disc diffusion method against four bacterial strains. Additionally, the volatile profiles of fennel hydrolates were determined by headspace solid-phase microextraction (HS-SPME) and GC-MS analysis. Fennel flowers gave the highest EO yield (1.95%), followed by mature fruits (1.43%), whereas significantly lower yields were obtained from leaves (0.69%) and stems (0.58%). *Trans*-anethole was identified as the dominant constituent (from 40.96% in stems to 80.71% in fruits), while *α*-phellandrene predominated in stem EO (42.77%). Hydrolate volatile profiles were more complex—particularly leaf hydrolate, where 29 compounds were identified. The principal constituents were *trans*-anethole (39.58–57.40%) and fenchone (16.01–28.80%), while the highest content of estragole was observed in fruit hydrolate (6.56%). The EOs demonstrated moderate antimicrobial activity, showing effectiveness exclusively against *Escherichia coli*, likely attributable to high phenylpropanoid (primarily *trans*-anethole) and fenchone contents.

## 1. Introduction

Fennel (*Foeniculum vulgare* Mill.) is an aromatic and medicinal plant species belonging to the Apiaceae family. Although native to the Mediterranean region, fennel is now widely distributed and cultivated in temperate and subtropical areas worldwide. All parts of the plant are edible and are traditionally used as culinary spices and herbal remedies [1].

Due to their distinctive aroma and diverse biological activities, essential oils (EOs) isolated from fennel umbels and fruits (commercially termed seeds) have been extensively characterised and studied [2,3,4]. In contrast, fewer studies have investigated EOs obtained from aerial parts [5,6] or particular plant parts, including leaves [7,8,9,10,11], stems [8], and rhizomes [12]. Furthermore, only a few studies compared EO profiles from different plant parts [8,11,13]. *trans*-Anethole is mainly reported as the major compound responsible for fennel EO properties, including anticarcinogenic, anti-inflammatory, antimicrobial, antioxidant, and insecticidal activities [3,6,7,8,9,10,13]. While *trans*-anethole is widely used in pharmaceutical applications, its less abundant *cis*-isomer is toxic to individuals [1]. Some studies reported that another phenylpropanoid present in fennel EO, estragole (methyl chavicol), acts also as a genotoxic and carcinogenic compound [14,15]. Therefore, the content of these compounds may represent a limiting factor for fennel EO applications [16].

Previous studies have shown that fennel EO yield, phytochemical profile, and bioactivity are cultivar-specific [17,18,19]. Variations in EO yield and chemical composition are influenced not only by plant physiology, but also by harvesting period and plant developmental stage [9,20,21,22]. Climatic conditions, particularly temperature and rainfall during the generative phase, play a crucial role in shaping EO profiles [2,23,24], as well as geographic location [16,25,26,27,28] and cultivation practice [18,29,30,31]. Despite extensive research, available data predominantly focus on cultivated fennel [32,33] or commercial EOs [34,35,36], while comprehensive investigations of wild-growing populations, particularly from the Mediterranean region, remain limited [8,9,10,11,13,29,37,38]. An overview of the major compounds in *Foeniculum vulgare* EOs from different plant parts and locations is presented in Table 1.

During EO distillation, polar volatiles may partition into the hydrolate, contributing to its aroma and biological properties. Hydrolates are increasingly recognised as by-products of EO production, with growing interest in their application in the food, pharmaceutical, and cosmetic industries [39,40]. However, data on fennel hydrolate composition remain scarce. In a hydrolate obtained from fennel seeds originating from the Czech Republic, estragole and fenchone accounted for nearly 60% of the composition, while the typically predominant *trans*-anethole was not detected [41].

In Croatia, fennel is widely distributed along the Adriatic coast and islands, thriving in dry, sunny habitats. Although previous findings indicate that volatile production is organ- and stage-dependent, no study has compared EO profiles among different plant parts of wild Croatian fennel. To date, only one study investigating the EO from Croatian fennel seeds [38] was reported. Several studies focused exclusively on EOs obtained from cultivated fennel seeds [21,42] or commercial EO samples [43]. Systematic research of non-cultivated Croatian fennel EOs has not been previously reported, as far as the authors know. Aside from thorough research of Croatian fennel EOs, this study also brings forth the chemical composition of fennel hydrolates. Hydrolates represent highly valuable yet largely overlooked by-products of EO distillation which remain poorly investigated in the available literature. To the best of our knowledge, this is the first report to comparatively assess hydrolates across different fennel aerial parts, thereby revealing intra-plant variability.

The present study therefore provides a comprehensive characterisation of volatiles (from EOs and hydrolates) obtained from different plant parts (stems, leaves, flowers, and fruits) of wild-grown *Foeniculum vulgare* collected from its natural habitat in Dalmatia, Croatia. In addition, the antimicrobial activity of Croatian wild-grown fennel EO is evaluated for the first time. The results offer novel insights into the differences in chemical profiles of the samples among the investigated plant parts as well as natural chemical diversity and potential chemotype differentiation of wild fennel populations shaped by local microclimatic conditions and/or adaptive metabolic responses.

## 2. Results and Discussion

### 2.1. Fennel Essential Oils

In this study EOs were isolated from different aerial parts of wild-grown Croatian fennel. Hydrodistillation was selected as an isolation technique, since Marčac et al. [42] reported a higher fennel EO yield compared to steam distillation, while gas chromatography–mass spectrometry (GC-MS) analysis showed no qualitative differences in chemical composition among the applied isolation procedures.

Fennel flowers (premature–waxy umbels), harvested while the herb was in full flowering, gave the highest EO yield (1.95%), followed by mature fruits (fully ripe umbels with seeds) with a slightly lower yield (1.43%). Šunić et al. [9] reported the highest EO yields for premature umbels at the waxy stage and fully ripe umbels in the early fruiting stage. The content of EO was lower in fully ripe seeds, while the lowest amount was detected in leaves. In accordance with the results for wild fennel from the Montenegro coast, these findings confirm that umbels can be harvested at the waxy stage to considerably reduce seed shedding and losses, as well as to increase EO yield [9]. In addition, Žutić et al. [21] highlighted the stage of “green seed” on the first-range umbel as suitable harvesting time due to the highest yield of EO along with the lowest yield of biomass. Much lower yields were observed for EOs from fennel leaves (0.69%) and stems (0.58%), similar to previous studies [8,9]. According to Aćimović et al. [23], the plant material investigated in the present study is classified as sweet fennel (2% yield; >80% *trans*-anethole, <10% estragole and 7.5% fenchone) compared to bitter fennel (4% yield; >60% *trans*-anethole and 15% fenchone). Higher EO yield (5.50%) was also observed from dried seeds of bitter fennel cultivated in Croatia [42]. Comparing two varieties of Croatian fennel, Žutić et al. [21] reported that var. *vulgare* gives a higher EO yield in all developmental stages. It should be noted that, in most reported studies [8,9,32,42], dry plant material was used, whereas the present study employed fresh fennel without previous pretreatment. Significant yield differences in seed samples were also observed based on geographic influences, for wild fennel seeds from 56 locations in Sicily (0.4–7.5%) [16], seeds from wild (3.67%) and domesticated (2.13%) plants from Morocco [29], seeds from three Tunisian (3.24–5.26%) and two French (3.81–4.12%) locations [32], and between fennel fruits cultivated in Egypt (1.32%) and Poland (4.14%) [33].

**Figure 1:**
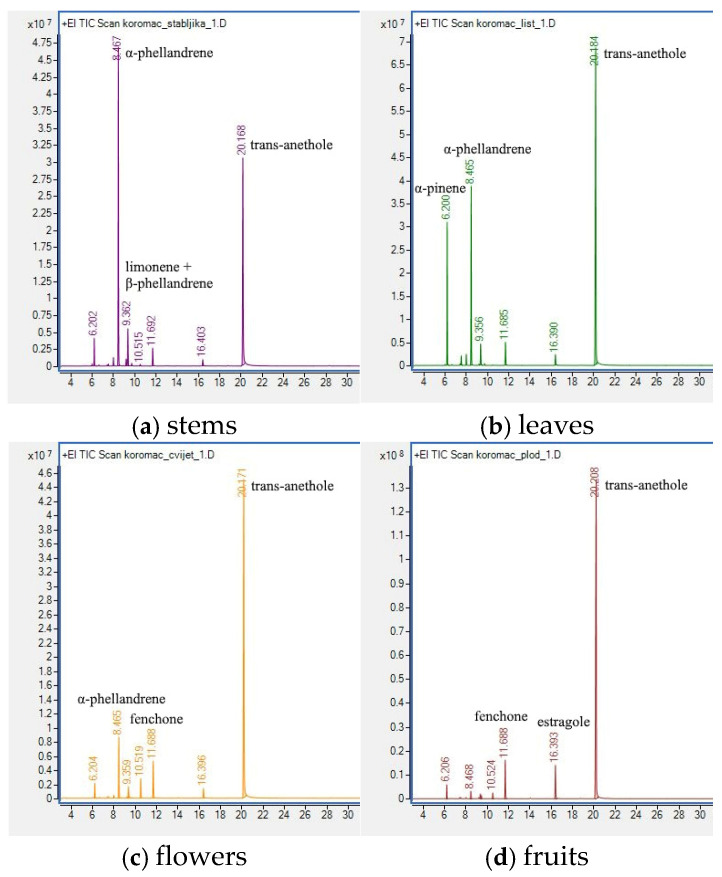
Total ion chromatograms (from 0 to 30 min) of fennel essential oils obtained from different plant parts.

The chemical composition of Croatian fennel EOs from different plant parts is presented in Table 2, while the corresponding total ion chromatograms are shown in Figure 1a–d.

**Figure 2:**
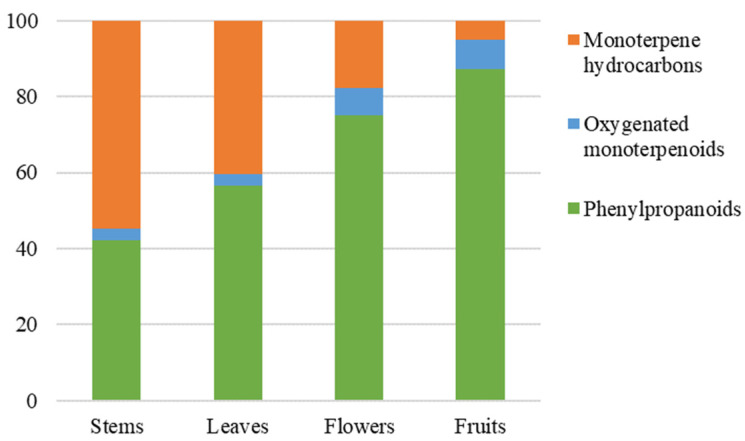
The main compound groups in fennel essential oils according to plant part.

As shown in Figure 2, the abundance of the main compound groups in fennel EOs varied markedly depending on the plant part. Monoterpene hydrocarbons were the dominant group in stems (54.87%) and remained abundant in leaves (40.28%), whereas their proportion substantially decreased in flower (17.59%) and fruits (4.96%). In contrast, phenylpropanoids exhibited the opposite trend, increasing progressively from stems (42.13%) and leaves (56.42%) to flowers (75.02%) and fruits (87.27%). Oxygenated monoterpenoids were present in lower amounts across all plant parts, ranging from 3.01% in stems to 7.78% in fruits, with a slightly higher level also observed in flower EO (7.24%). Overall, the results indicate a clear shift from monoterpene hydrocarbon-rich profiles in vegetative parts toward phenylpropanoid-dominated profiles in reproductive organs.

**Figure 3:**
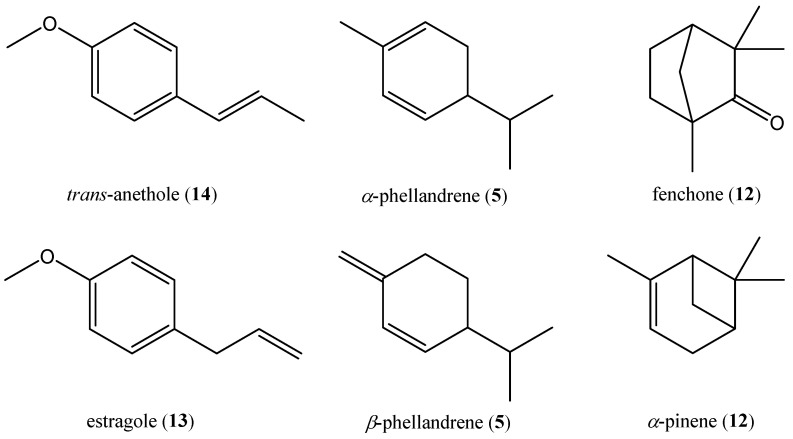
Structures of the most abundant components in wild Croatian fennel essential oil.

The main component in leaf, flower and fruit EOs was *trans*-anethole (**14**) with content of 54.77%, 72.94% and 80.71%, respectively. In contrast, *α*-phellandrene (**5**) was the main component in the stem EO (42.77%) and its content decreased in the leaves (20.78%) and flowers (9.49%), while in fruits it was only 1.19%. Compared to *α*-phellandrene, the same trend with considerably lower contents (0.86–5.64%) was observed for *β*-phellandrene (**8**). However, the signal at 9.36 min was not pure and represented a mixture of *β*-phellandrene and limonene (**7**), two cyclic monoterpenes with similar boiling points that are difficult to separate on used chromatographic column. As for the other monoterpene hydrocarbons, *α*-pinene (**2**) was found in all fennel EOs (from 1.88% for the fruits to 14.31% for the leaves), while *β*-pinene (**3**) was revealed in the EO distilled from the leaves (1.08%) and stems (0.27%). In addition, *β*-myrcene (**4**) was identified in the EOs from the leaves (1.28%), stems (1.12%), and flowers (0.52%), while *γ*-terpinene (**11**) was detected in the EOs from the flowers (3.39%), fruits (1.03%), and stems (0.29%). Moreover, *p*-cymene (**6**), *β*-ocimene (**10**), and *α*-thujene (**1**) contents in the fennel stem EO were 0.96%, 0.40% and 0.23%, respectively, while in other EOs these compounds were present only in trace amounts. Another phenylpropanoid identified in the fennel EOs was estragole (**13**), whose content was notably lower (compared to *trans*-anethole) but also increased from the stems (1.17%) to leaves (1.65%), flowers (2.08%), and fruits (6.56%). Structures of the most abundant components in the wild Croatian fennel EOs are shown in Figure 3. One of them is fenchone (**12**) with the same content (7.24%) in the EOs from the flowers and fruits and lower contents from leaves and stems (3.30% and 3.01%, respectively). Another oxygenated monoterpenoid, 1,8-cineole, also known as eucalyptol (**9**), was identified only in the fennel fruit EO (0.54%).

Relative contents (%) calculated from peak areas revealed significant differences among plant parts of wild-growing Croatian fennel for all identified compounds, indicating that the chemical composition of fennel EO depends on the plant organ. The investigated EOs belong to a *trans*-anethole-rich chemotype, as *trans*-anethole predominates and is responsible for the characteristic sweet aroma [17]. Its content consistently increases toward the generative organs. In contrast, monoterpene hydrocarbons, particularly *α*-phellandrene, decrease toward the fruit EO.

Similar results regarding the dominance of *trans*-anethole in Croatian fennel seed EO were also reported by Politeo et al. [38], who detected this compound at 77.6%. Damjanović et al. [37] also reported high contents of *trans*-anethole (62.0%) and fenchone (20.3%) in EOs from wild-growing fennel seeds native to southern Montenegro. High *trans*-anethole content was also reported in Greek (81.50%) and Turkish (82.30%) seeds by Chatzopoulou et al. [18], while another study reported that the main components of EOs obtained from Turkish seeds were *trans*-anethole (81.55%), limonene (5.88%), and estragole (4.75%) [4]. In contrast, studies on fennel samples from North African Mediterranean countries reported the dominance of estragole, fenchone, and limonene. Ahmed et al. [28] detected estragole (51.04%), limonene (11.45%), and fenchone (8.19%) in Egyptian samples, while the *trans*-anethole content was significantly lower (3.62%). In the study by Khammassi et al. [44] on several Tunisian wild fennel seed samples, the main compounds were estragole (66.09–85.23%), fenchone (5.18–23.09%), and limonene (4.3–10.25%). In Moroccan wild seeds, the main compounds were anethole (52.27%), estragole (35.33%), and fenchone (4.32%) [29]. Sicilian samples showed high estragole content (34–89%), while (*E*)-anethole was the second dominant phenylpropanoid (0.1–36%), suggesting the presence of an African chemotype among those populations [16]. Although fennel seeds have been extensively studied, the other parts of the plant remain insufficiently investigated.

Regarding other plant organs, Šunić et al. [9] reported only minor percentage differences and some specific components among umbel developmental stages in Montenegrin fennel. Consistent with the present results for Croatian fennel flower, *trans*-anethole, fenchone, and *α*-phellandrene were the dominant constituents. However, studies on Turkish fennel flowers reported a different profile, with estragole (38.9%), fenchone (19.4%), *γ*-terpinene (9.0%), and α-terpinyl acetate (8.8%) as major components [13]. Similarly, Egyptian umbels were characterised by estragole, anethole, limonene, fenchone, and *γ*-terpinene, with percentages of 51.18%, 25.08%, 12.22%, 6.57%, and 2.86%, respectively [11].

Leaf EOs exhibit even greater variability. Tunisian samples were rich in monoterpene hydrocarbons (41.56–63.13%) and phenylpropanoids (35.87–55.29%), with limonene (8.40–50.34%) and estragole (35.87–54.28%) being dominant constituents [10]. Egyptian leaf EO contained anethole (37.94%), estragole (35.56%), limonene (17.46%), *trans*-*β*-ocimene (1.53%), and fenchone (1.49%) [11], while Turkish leaf samples were rich in estragole (51.7%), limonene (11.5%), terpinolene (10.5%), and fenchyl acetate (6.6%) [13]. In contrast, Šunić et al. [9] reported that EO obtained from leaves collected in Montenegro contained (*E*)-anethole (32.5%), *α*-phellandrene (18.8%), *p*-cymene (17.3%), and *β*-phellandrene (10.3%) as the main compounds. In another Montenegrin coastal sample, the most abundant compounds were (*E*)-anethole (51.4%) and estragole (9.3%) [8].

Stem EO from the same Montenegrin region was similarly dominated by (*E*)-anethole (55.7%) and estragole (7.8%) [8]. Turkish stem EO showed a markedly different profile, with fenchyl acetate (35.3%), limonene (26.8%), *trans*-limonene oxide (8.5%), and *endo*-fenchyl acetate (4.6%) as major constituents [13].

Overall, the present results confirm pronounced qualitative and quantitative differences in EO composition among plant parts and geographical origins. Croatian fennel shows greater similarity to neighbouring Mediterranean populations characterised by *trans*-anethole dominance, in contrast to the distinct estragole-rich chemotypes reported for North African samples. Such variability further underscores the significant influence of both biotic and abiotic factors on EO composition.

### 2.2. Fennel Hydrolates

**Figure 4:**
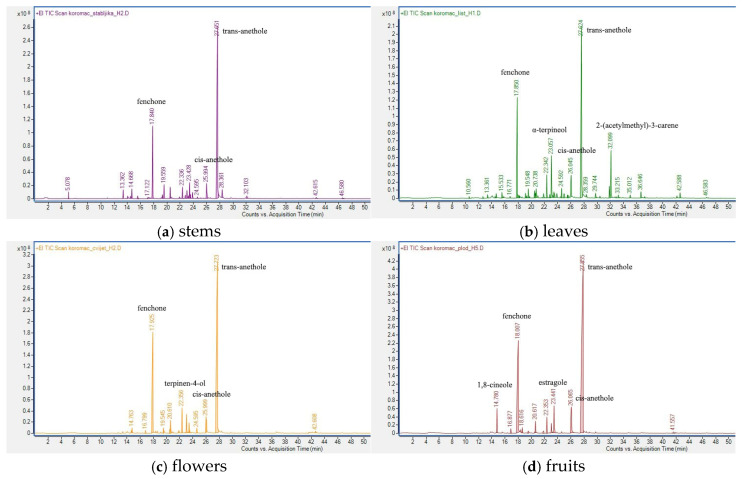
Total ion chromatograms (from 0 to 50 min) of fennel hydrolates obtained from different plant parts.

The chemical composition of fennel hydrolates from different plant parts is presented in Table 3, while the corresponding total ion chromatograms are shown in Figure 4a–d.

**Figure 5:**
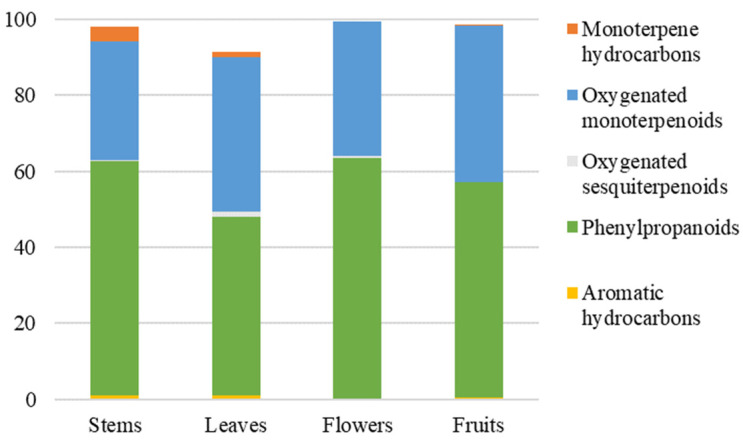
The main compound groups in fennel hydrolates according to plant part.

Compared with fennel EOs, the volatile profiles of hydrolates were more complex, with a higher number of identified constituents. In total, 29 compounds were identified in fennel leaf hydrolate, followed by hydrolates obtained from stems (22 compounds), fruits (19 compounds), and flowers (15 compounds). The abundance of the main compound groups in fennel hydrolates also varied depending on the plant part. As shown in Figure 5, a predominance of phenylpropanoids and oxygenated monoterpenoids was observed. Phenylpropanoids were the dominant group in all fennel hydrolates, showing considerable variations among plant parts and ranging from leaves (46.99%) and fruits (56.83%) to stems (61.66%), and reaching a maximum in flowers (63.41%). Oxygenated monoterpenoids were also highly abundant, ranging from 30.97% in stems to 40.72% in leaves and 40.96% in fruits. Monoterpene hydrocarbons, which were predominant in stem EO, also showed slightly higher content in stem hydrolate (4.07%). Their content was lower in leaves (1.26%) and fruits (0.44%), while they were absent in flower hydrolate. Oxygenated sesquiterpenoids were minor constituents in leaf (1.24%), flower (0.51%), and stem (0.32%) samples. Aromatic hydrocarbons were present in stems and leaves (about 1%), as well as in fruit hydrolate (0.41%).

The main components in all fennel hydrolates were *trans*-anethole (**29**) and fenchone (**10**) with relative contents ranging from 39.58 to 57.40% and from 16.01 to 28.80%, respectively. In addition, *cis*-anethole (**27**) was also found in all samples (2.53–5.07%), while the highest content of estragole (**24**) was observed in the fruit hydrolate (6.56%). Carvacrol (**30**) and *trans*-methyl isoeugenol (**33**) showed the highest content in the hydrolates obtained from stems (1.80%) and leaves (1.15%), respectively, while *p*-anisaldehyde (**28**) was found in the fruit hydrolate (1.02%). Unlike fennel EOs, monoterpene hydrocarbons *α*- and *β*-phellandrene (**3**, **6**) were present in low amounts, while *α*-pinene was not detected. Oxygenated monoterpenoids *p*-menth-2-en-1-ol (**14**, **16**) and piperitol (**23**, **25**) were mostly found in the stem hydrolate, and their contents consistently decreased toward the generative organs. In contrast, the content of camphor (**18**) increased and reached the maximum for the fruit hydrolate (2.51%) as did 1,8-cineole (**7**; 4.09%). The hydrolate obtained from fennel leaves is characterised by high contents of 2-(acetylmethyl)-3-carene (**31**), *α*-terpineol (**22**), *cis*-verbenol (**17**), and *trans*-3-caren-2-ol (**26**). The relative content of terpinen-4-ol (**20**) was around 3% in all fennel hydrolates. For the fruit hydrolate, the signal at 15.52 min represented a mixture of *γ*-terpinene and benzeneacetaldehyde (**8**).

During optimisation of headspace solid-phase microextraction (HS-SPME), subsequent GC-MS analysis showed good repeatability of retention times, while the relative peak areas deviated significantly. Although it was hard to consistently and reproducibly extract compounds from hydrolates onto SPME fibre, two analyses were successfully performed for each fennel hydrolate. However, for more precise quantification, calibration curves for each component, or at least for the most abundant ones, are required.

Compared with the study by Šilha et al. [41], hydrolates obtained as by-products during the hydrodistillation of wild Croatian fennel showed a different chemical composition, as expected. Hydrolate obtained from seeds contained a total of 13 compounds. Estragole and fenchone were the main components (33.0 and 26.5%, respectively), while *trans*-anethole was not detected. These results suggest that fennel from the reported study represents a different chemotype compared to *trans*-anethole-rich chemotype investigated in the present work. Owing to the antimicrobial activity reported, future research should focus on the bioactivity of Croatian fennel hydrolates. Since higher antimicrobial activity was observed for concentrated hydrolates, solid-phase extraction (SPE) is recommended for the sample pretreatment [41].

### 2.3. Antimicrobial Activity

Numerous studies have investigated the bioactivity of fennel EOs, including antioxidant [28,45], antibacterial [35,45], antifungal [4,33], and cytotoxic [6] effects. Variations in antimicrobial activity have frequently been linked to differences in chemical composition, particularly the ratios of *trans*-anethole, estragole, and fenchone [13,29], as well as to minor constituents that may act synergistically [7,46].

The antimicrobial activity of Croatian fennel EOs was not evaluated in previous studies. Therefore, EOs from different plant parts were individually tested against pathogenic bacteria *Staphylococcus aureus* ATCC 25923, *Bacillus cereus* ATCC 14579, and *Escherichia coli* ATCC 25922, as well as against the spoilage bacterium *Pseudomonas aeruginosa* ATCC 27853. Disc diffusion assay showed moderate antimicrobial activity of fennel EOs only against Gram-negative *E. coli*. The results are presented in Table 4. The largest diameter of the inhibition zone was observed for the fennel fruit EO (14.3 mm), followed by the fennel flower EO (9.7 mm) which may be due to their higher content of phenylpropanoids, mainly *trans*-anethole, as well as increased fenchone content. Similarly, Wodnicka et al. [33] reported that a fennel EO chemotype with *trans*-anethole and fenchone as the main components showed better antimicrobial activity. In contrast, monoterpene hydrocarbons, such as *α*-phellandrene (the predominant compound in the stem EO) and *α*-pinene (abundant in the leaf EO) were associated with considerably weaker antimicrobial activity. Hence, similar inhibition zones were detected for the EOs obtained from fennel leaves and stems (7.7 and 7.2 mm, respectively). For Gram-positive bacteria, including *S. aureus* and *B. cereus*, as well as Gram-negative bacterium *P. aeruginosa*, the inhibition zone did not exceed 6.5 mm in diameter, indicating a lack of notable antimicrobial activity. The different susceptibility of *E. coli* compared to *P. aeruginosa* suggests that antimicrobial activity depends not only on Gram classification but also on strain-specific membrane characteristics and resistance mechanisms [47].

A previous study on the EO obtained from cultivated fennel seeds also identified *E. coli* as the most sensitive bacterium among those tested, as well as identifying the high resistance of *P. aeruginosa* and *B. cereus* [29]. Furthermore, investigation of the antibacterial and biofilm-inhibiting effects of fennel honey and EO, individually and in combination, showed that *E. coli* was the most sensitive microorganism, while *P. aeruginosa* was the most resistant [46]. On the other hand, Anwar et al. [45] reported appreciable antimicrobial activity of the fennel EO from Pakistan with *B. subtilis* being the most sensitive microorganism, while *E. coli* showed the lowest susceptibility. Elkiran and Telhuner [13] reported that Gram-positive bacteria were more sensitive to fennel EOs from different aerial parts of the plant. The highest effect was obtained for the fennel seed EO against *S. aureus* with a 30 mm inhibition zone, while among Gram-negative species the best results are also shown against *E. coli*. However, direct comparison of antimicrobial activity between presented results and those of previous studies should be approached with caution due to differences in chemical composition discussed above. Furthermore, disc diffusion assays provide only preliminary insight into the antimicrobial potential of Croatian fennel EOs.

## 3. Materials and Methods

### 3.1. Plant Material

**Figure 6:**
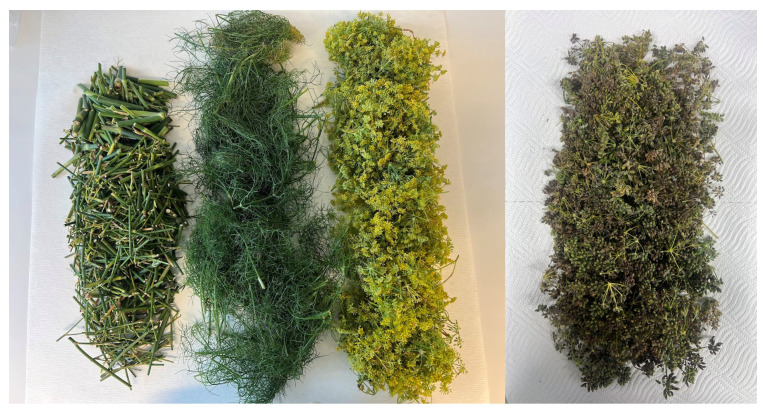
Stems, leaves, flowers, and fruits of *Foeniculum vulgare* Mill.

Different aerial parts of wild fennel (*Foeniculum vulgare* Mill.) were collected in the hinterland of Split, Croatia (43.46° N, 16.70° E), in August 2025 (stems, leaves, and flowers) and October 2025 (fruits). After harvesting, the fresh plant material was cleaned, and the separated plant parts (Figure 6) were stored at −18 °C until EOs and hydrolates were isolated. A voucher specimen of plant material (AVFV1) was deposited in the herbarium of the University of Split, Faculty of Chemistry and Technology.

### 3.2. Isolation of Essential Oils and Hydrolates—Hydrodistillation

Fennel EOs and hydrolates were isolated by hydrodistillation using a modified Clevenger-type apparatus [48]. Approximately 100 ± 5 g of each plant part (stems, leaves, flowers, and fruits), covered with 1.5 L of water, were placed in a round-bottom flask and distilled for 3 h. A mixture of pentane and diethyl ether (2:1, *v*/*v*) was used as a trap for the EOs. Anhydrous sodium sulphate was added to remove water from the collected EOs. The solvent trap was carefully evaporated with a gentle steam of nitrogen to constant mass in order to obtain pure EOs. The EO yield was calculated as the mass of EO obtained from a defined amount of fresh plant material (*w*/*w*). For GC-MS analysis 1 µL of each EO was dissolved in 1 mL of hexane (1:1000, *v*/*v*). The aqueous layers beneath the solvent trap, representing fennel hydrolates, were also collected for subsequent headspace solid-phase microextraction (HS-SPME).

### 3.3. GC-MS Analysis of Essential Oils

Chemical composition of fennel EOs was determined using the 8890 GC System equipped with 7693A Autosampler and 7000D GC/TQ tandem mass spectrometer (Agilent Inc., Santa Clara, CA, USA). Chromatographic separation was performed on the HP-5MS UI column (30 m × 0.25 mm i.d., 0.25 μm film thickness; Agilent Inc.). The carrier gas was helium (grade 5.0) and the flow rate was constant at 1.0 mL/min. The inlet temperature was set to 250 °C and 1 µL of EO diluted in hexane (1:1000, *v*/*v*) was injected in split mode (50:1) using a 5190-2295 liner (Agilent Inc.). The initial oven temperature of 60 °C with a hold time of 2 min was ramped to 246 °C at 3 °C/min and held for 25 min. The ionisation energy was set to 70 eV, while the MS transfer line, the ion source, and the quadrupole temperatures were 280 °C, 230 °C, and 150 °C, respectively. The analyses were performed in full-scan MS mode (*m*/*z* 40–450). The run time was 89 min including a solvent delay of 3 min. The *n*-alkane standard (C7–C40; Supelco Inc., Sigma-Aldrich, Bellefonte, PA, USA) was analysed under the same conditions to calculate retention indices (RIs) for each EO constituent and compare them with the literature data. Additionally, available standards (*α*- and *β*-pinene, *p*-cymene, limonene, 1,8-cineole, and fenchone) were analysed under the same conditions as the EO samples to confirm their presence in fennel EOs. Data acquisition and processing were performed using MassHunter Workstation Software (version 10.0; Agilent Inc.). Compound identification was based on a software comparison of mass spectra (MS) with those from databases NIST 17 (Gaithersburg, MD, USA) and Wiley 9N08 (New York, NY, USA). All analyses were done in triplicate and the relative content (%) of each compound, calculated from peak areas, was expressed as the mean value ± standard deviation.

### 3.4. HS-SPME-GC-MS Analysis of Hydrolates

The solid-phase microextraction of volatiles from fennel hydrolates was performed using an SPME fibre with DVB/CAR/PDMS coating (50/30 μm; Supelco Inc., Sigma-Aldrich, Bellefonte, PA, USA). Before use, the fibre was conditioned in the GC inlet at 250 °C for 1 h. The fennel hydrolate (2 mL) was transferred to a glass vial and a small amount of solid NaCl was added. The vial was then sealed with a magnetic cap and the SPME fibre was inserted into the headspace. The hydrolate sample was equilibrated at 50 °C under continuous stirring for 40 min. During that period the fibre was exposed to the hydrolate headspace to allow adsorption of volatiles. After extraction, the SPME fibre was transferred to the GC inlet at 250 °C, where thermal desorption was performed for 3 min using a 5190-4048 liner (Agilent Inc.). The chemical composition of fennel hydrolates was determined by GC-MS using the same instrument and column as for EOs analysis. The method conditions were the same as described above with the exception of the oven temperature program. The column temperature was 40 °C for 3 min and then increased at a rate of 3 °C/min to 250 °C, where it was held for 2 min. The run time was 75 min, with no solvent delay applied. HS-SPME-GC-MS analysis of *n*-alkane standard (C7–C40; Supelco Inc.) was conducted under the same conditions to calculate retention indices (RIs). Each hydrolate analysis was performed in duplicate and the relative content (%) of each compound was expressed as the mean value ± standard deviation.

### 3.5. Antimicrobial Activity Determination—Disc Diffusion Method

The protocol used to determine the antimicrobial activity of the fennel EOs was previously described by the European Committee on Antimicrobial Susceptibility Testing (EUCAST) [49]. The biological activity of the EOs was tested against *Staphylococcus aureus* ATCC 25923, *Bacillus cereus* ATCC 14579, *Escherichia coli* ATCC 25922, and *Pseudomonas aeruginosa* ATCC 27853. An overnight bacterial inoculum (14–16 h, 37 °C) was prepared by transferring one bacterial colony from Mueller–Hinton Agar (MHA) to 5 mL of Mueller–Hinton Broth (MHB). Then, 3 mL of the prepared bacterial inoculum was added to 27 mL of MHB and incubated for 1–1.5 h, and optical density was adjusted to 0.5. A working inoculum (1–2 × 10^5^ colony forming unit (CFU)/mL) was prepared by adding 38 mL of MHB to 2 mL of bacterial inoculum. The concentration of the working bacterial inoculum was confirmed using the serial dilution method by Koch as previously described [50]. Petri plates containing 20 mL MHA were inoculated by spreading the working bacterial inoculum over the entire surface using sterile cotton swabs rolled in the inoculum. A volume of 10 µL of the fennel EO samples was added to filter paper discs 6 mm in diameter and placed on the agar surface. Antibiotic gentamicin was used as a positive control, with inhibition zones within the range proposed by EUCAST [51]. The Petri plates were incubated at 37 °C for 24 h, and the zone of inhibition was measured in millimetres and recorded when the diameter was greater than 6.5 mm.

### 3.6. Statistical Analysis

Data analysis was performed using Microsoft Excel and SPSS software (version 25.0; IBM Corp., New York, NY, USA). Differences in the percentage composition of fennel EO constituents among plant parts were assessed using one-way analysis of variance (ANOVA), followed by Tukey’s post hoc test, with significance set at *p* < 0.05. The same statistical analysis was applied to disc diameter measurements from antimicrobial activity assays. As hydrolate analyses were performed in duplicate, statistical power for these data was limited, and hydrolate results were evaluated using descriptive statistics only.

## 4. Conclusions

Variations in fennel EO yield and chemical composition are influenced by plant variety, climatic conditions, geographic influences, as well as plant physiology and developmental stage. The present study provides a comprehensive characterisation of EOs and hydrolates obtained from different plant parts (stems, leaves, flowers, and fruits) of Croatian wild-grown *F. vulgare* collected from its natural habitat.

The differences in EOs yield and volatile profiles point to intra-plant variability. Monoterpene hydrocarbons were present in high amounts in vegetative plant parts (especially *α*-phellandrene in stems), while in generative plant parts (flowers and fruits) the dominance of phenylpropanoids, namely *trans*-anethole (73 and 81%) was observed. Moreover, the increasingly recognised by-products of EO production (hydrolates) were characterised and confirmed previous findings, with *trans*-anethole and fenchone being dominant in all samples. The fennel fruit and flower EOs exhibited moderate antimicrobial activity against *E. coli*, which may also be attributed to the higher amounts of *trans*-anethole and fenchone in these samples compared to others. The present findings provide chemotype-specific data on the wild Croatian fennel and highlight EOs obtained from fruits and flowers as promising candidates for further investigation in food preservation, phytopharmaceutical, and synergistic antimicrobial applications.

## Figures and Tables

**Table 1:** Overview of the literature on the dominant compounds in *Foeniculum vulgare* essential oils according to plant part and geographic origin.

Plant Part	Location	Major Compounds (%)	Ref.
seeds	Turkey	*trans*-anethole (81.55%), limonene (5.88%), estragole (4.75%)	[4]
	Greece ^1^	anethole (64.30–82.30%), fenchone (0.90–20.60%), estragole (2.60–4.00%), limonene (3.30–7.20%)	[18]
	Egypt	estragole (51.04%), limonene (11.45%), fenchone (8.19%), *trans*-anethole (3.62%)	[28]
	Morocco	anethole (52.27%), estragole (35.33%), fenchone (4.32%), *α*-pinene (2.01%)	[29]
	Montenegro	*trans*-anethole (62.0%), fenchone (20.3%), estragole (4.90%), limonene (3.15%), *α*-pinene (2.81%)	[37]
	Croatia	*trans*-anethole (77.6%), fenchone (12.4%), estragole (2.2%)	[38]
seeds	Turkey	estragole (53.3%), fenchone (24.5%), *trans*-*β*-terpineol (5.1%), *α*-pinene (3.3%)	[13]
flowers		estragole (38.9%), fenchone (19.4%), *γ*-terpinene (9.0%), *α*-terpinyl acetate (8.8%)	
leaves		estragole (51.7%), limonene (11.5%), terpinolene (10.5%), fenchyl acetate (6.6%), *α*-phellandrene (6.1%)	
stems		fenchyl acetate (35.3%), limonene (26.8%), *trans*-limonene oxide (8.5%), *endo*-fenchyl acetate (4.6%)	
umbels	Egypt	estragole (51.18%), anethole (25.08%), limonene (12.22%), fenchone (6.57%)	[11]
leaves		anethole (37.94%), estragole (35.56%), limonene (17.46%)	
leaves	Tunisia ^2^	estragole (35.87–54.28%), limonene (8.40–50.34%), *α*-thujene (1.03–18.8%), *β*-myrcene (0.68–13.2%)	[10]
umbels ^3^	Montenegro	*trans*-anethole (64.0–75.5%), fenchone (4.8–13.7%), estragole (2.1–10.3%), *α*-phellandrene (1.1–11.0%)	[9]
leaves		*trans*-anethole (32.5%), *α*-phellandrene (18.8%), *p*-cymene (17.3%), *β*-phellandrene (10.3%)	[9]
leaves		*trans*-anethole (51.4%), estragole (9.3%), *p*-cymene (6.5%), *α*-phellandrene (5.9%), *β*-phellandrene (4.9%)	[8]
stems		*trans*-anethole (55.7%), estragole (7.8%), *p*-cymene (3.9%), *cis*-thujone (3.7%), ledol (3.0%)	[8]
rhizome	Italy	*trans*-anethole (85.59%), limonene (5.97%), *exo*-fenchyl acetate (3.32%), estragole (1.91%)	[12]

^1^ Cultivars from Greece, Turkey, and Bulgaria. ^2^ Wild from 12 locations. ^3^ 4 maturation stages.

**Table 2:** Chemical composition of fennel essential oils from different plant parts.

No.	Compound	*R*t (min)	RI_exp._	RI_lit._	Content (%)				Identification
					Stems	Leaves	Flowers	Fruits	
**1**	*α*-thujene	6.00 ± 0.005	930	930	0.23 ± 0.01	tr	tr	tr	MS, RI
**2**	*α*-pinene	6.20 ± 0.002	937	939	3.19 ± 0.01 b	14.31 ± 0.06 a	2.10 ± 0.05 c	1.88 ± 0.03 d	MS, RI, st
**3**	*β*-pinene	7.53 ± 0.002	979	979	0.27 ± 0.02 b	1.08 ± 0.02 a	tr	tr	MS, RI, st
**4**	*β*-myrcene	8.00 ± 0.003	992	991	1.12 ± 0.03 b	1.28 ± 0.02 a	0.52 ± 0.04 c	tr	MS, RI
**5**	*α*-phellandrene	8.47 ± 0.002	1006	1002	**42.77 ± 0.08** a	20.78 ± 0.02 b	9.49 ± 0.03 c	1.19 ± 0.02 d	MS, RI
**6**	*p*-cymene	9.20 ± 0.002	1027	1024	0.96 ± 0.04	tr	tr	tr	MS, RI, st
**7**	limonene	9.36 ± 0.004	1031	1029	5.64 ± 0.07 a	2.83 ± 0.04 b	2.09 ± 0.02 c	0.86 ± 0.03 d	MS, RI, st
**8**	*β*-phellandrene								MS, RI
**9**	1,8-cineole	9.46 ± 0.002	1034	1031	tr	tr	tr	0.54 ± 0.01	MS, RI, st
**10**	*β*-ocimene	9.71 ± 0.009	1041	1037	0.40 ± 0.02	tr	tr	tr	MS, RI
**11**	*γ*-terpinene	10.52 ± 0.004	1062	1059	0.29 ± 0.01 c	tr	3.39 ± 0.06 a	1.03 ± 0.01 b	MS, RI
**12**	fenchone	11.69 ± 0.003	1089	1086	3.01 ± 0.03 c	3.30 ± 0.04 b	7.24 ± 0.01 a	7.24 ± 0.01 a	MS, RI, st
**13**	estragole	16.39 ± 0.004	1198	1196	1.17 ± 0.10 d	1.65 ± 0.11 c	2.08 ± 0.07 b	6.56 ± 0.07 a	MS, RI
**14**	*trans*-anethole	20.18 ± 0.018	1287	1284	**40.96 ± 0.22** d	**54.77 ± 0.11** c	**72.94 ± 0.22** b	**80.71 ± 0.14** a	MS, RI
	Monoterpene hydrocarbons (**1–8**, **10**, **11**)				54.87	40.28	17.59	4.96	
	Oxygenated monoterpenoids (**9**, **12**)				3.01	3.30	7.24	7.78	
	Phenylpropanoids (**13**, **14**)				42.13	56.42	75.02	87.27	
	**Total identified:**				**100.01**	**100.00**	**99.85**	**100.01**	
	**EO yield** (g EO/100 g fresh plant material)				0.58	0.69	1.95	1.43	

*R*t—retention time; RI_exp._—experimental retention index; RI_lit._—literature retention index; MS—mass spectra; st—standard compound; tr—in traces. Values with different letters (a–d) within the same row differ significantly (*p* < 0.05). Each analysis was performed in triplicate (*n* = 3).

**Table 3:** Chemical composition of fennel hydrolates from different plant parts.

No.	Compound	*R*t (min)	RI_exp._	RI_lit._	Content (%)				Identification
					Stems	Leaves	Flowers	Fruits	
**1**	2,4-thujadiene	10.54 ± 0.033	930	956	tr	0.22 ± 0.01	tr	tr	MS
**2**	2,3-dehydro-1,8-cineole	12.63 ± 0.022	975	986	tr	0.22 ± 0.01	tr	tr	MS, RI
**3**	*α*-phellandrene	13.35 ± 0.053	988	1002	1.51 ± 0.14	0.48 ± 0.00	tr	0.21 ± 0.01	MS, RI
**4**	α-terpinene	14.05 ± 0.073	1001	1017	0.47 ± 0.03	tr	tr	0.23 ± 0.01	MS, RI
**5**	*p*-cymene	14.45 ± 0.065	1010	1024	0.41 ± 0.01	tr	tr	tr	MS, RI
**6**	*β*-phellandrene	14.64 ± 0.043	1015	1029	1.68 ± 0.13	0.56 ± 0.04	tr	tr	MS, RI
**7**	1,8-cineole	14.90 ± 0.161	1021	1031	tr	tr	0.63 ± 0.06	4.09 ± 0.00	MS, RI
**8**	benzeneacetaldehyde	15.52 ± 0.038	1034	1042	0.64 ± 0.09	0.83 ± 0.04	tr	0.41 ± 0.00 *	MS, RI
**9**	*cis*-sabinene hydrate	16.78 ± 0.022	1060	1070	tr	0.24 ± 0.02	0.42 ± 0.04	tr	MS, RI
**10**	fenchone	18.03 ± 0.317	1084	1086	**16.40 ± 2.20**	**16.01 ± 0.43**	**24.61 ± 2.11**	**28.80 ± 1.90**	MS, RI
**11**	*endo*-fenchol	19.11 ± 0.012	1105	1116	tr	0.58 ± 0.04	tr	tr	MS, RI
**12**	linalool	19.24 ± 0.048	1108	1096	tr	tr	tr	0.49 ± 0.06	MS, RI
**13**	*exo*-fenchol	19.28 ± 0.025	1108	1121	0.72 ± 0.08	0.21 ± 0.00	tr	tr	MS, RI
**14**	*cis*-*p*-menth-2-en-1-ol	19.65 ± 0.201	1117	1121	2.69 ± 0.43	1.14 ± 0.09	0.77 ± 0.07	0.33 ± 0.02	MS, RI
**15**	*trans*-2-pinanol	19.93 ± 0.033	1123	1137	tr	tr	tr	0.46 ± 0.03	MS, RI
**16**	*trans*-*p*-menth-2-en-1-ol	20.48 ± 0.014	1135	1140	2.10 ± 0.23	0.96 ± 0.02	0.51 ± 0.01	tr	MS, RI
**17**	*cis*-verbenol	20.73 ± 0.008	1140	1141	tr	1.28 ± 0.04	tr	tr	MS, RI
**18**	camphor	20.75 ± 0.214	1141	1146	tr	0.70 ± 0.04	1.64 ± 0.18	2.51 ± 0.01	MS, RI
**19**	*δ*-terpineol	22.23 ± 0.025	1171	1166	tr	tr	tr	0.18 ± 0.00	MS, RI
**20**	terpinen-4-ol	22.44 ± 0.189	1175	1177	2.09 ± 0.33	2.93 ± 0.03	3.23 ± 0.31	2.72 ± 0.08	MS, RI
**21**	*p*-cymen-8-ol	22.79 ± 0.014	1182	1182	0.68 ± 0.02	0.52 ± 0.04	tr	tr	MS, RI
**22**	*α*-terpineol	23.12 ± 0.157	1188	1188	1.58 ± 0.14	6.31 ± 0.14	2.50 ± 0.14	0.82 ± 0.12	MS, RI
**23**	*cis*-piperitol	23.23 ± 0.011	1190	1196	0.48 ± 0.04	0.26 ± 0.03	tr	tr	MS, RI
**24**	estragole	23.47 ± 0.084	1195	1196	2.61 ± 0.01	0.72 ± 0.06	1.43 ± 0.15	6.56 ± 0.31	MS, RI
**25**	*trans*-piperitol	23.86 ± 0.011	1203	1208	1.31 ± 0.23	0.74 ± 0.06	tr	tr	MS, RI
**26**	*trans*-3-caren-2-ol	24.62 ± 0.088	1221	-	0.36 ± 0.04	1.15 ± 0.07	0.76 ± 0.01	0.23 ± 0.01	MS
**27**	*cis*-anethole	26.07 ± 0.108	1253	1252	3.28 ± 0.43	5.07 ± 0.70	4.12 ± 0.78	2.53 ± 0.01	MS, RI
**28**	*p*-anisaldehyde	27.23 ± 0.166	1277	1277	tr	tr	tr	1.02 ± 0.11	MS, RI
**29**	*trans*-anethole	27.89 ± 0.407	1291	1284	**55.77 ± 3.39**	**39.58 ± 0.97**	**57.40 ± 1.40**	**46.54 ± 1.99**	MS, RI
**30**	carvacrol	28.51 ± 0.284	1304	1299	1.80 ± 0.21	0.42 ± 0.01	0.31 ± 0.01	0.35 ± 0.04	MS, RI
**31**	2-(acetylmethyl)-3-carene	32.10 ± 0.009	1388	1380	0.76 ± 0.12	7.05 ± 0.02	tr	tr	MS, RI
**32**	*cis*-methyl isoeugenol	35.01 ± 0.002	1459	1453	tr	0.47 ± 0.01	tr	tr	MS, RI
**33**	*trans*-methyl isoeugenol	36.66 ± 0.014	1498	1492	tr	1.15 ± 0.01	0.46 ± 0.05	0.18 ± 0.01	MS, RI
**34**	*τ*-muurolol	42.11 ± 0.000	-	1642	tr	0.40 ± 0.01	tr	tr	MS
**35**	*α*-cadinol	42.60 ± 0.014	-	1646	0.32 ± 0.01	0.84 ± 0.05	0.51 ± 0.12	tr	MS
**36**	benzyl benzoate	46.57 ± 0.030	-	1760	0.41 ± 0.01	0.23 ± 0.01	tr	tr	MS
	Monoterpene hydrocarbons (**1**, **3–6**)				4.07	1.26	-	0.44	
	Oxygenated monoterpenoids (**2**, **7**, **9–23**, **25**, **26**, **30**, **31**)				30.97	40.72	35.38	40.98	
	Oxygenated sesquiterpenoids (**34**, **35**)				0.32	1.24	0.51	-	
	Phenylpropanoids (**24**, **27–29**, **32**, **33**)				61.66	46.99	63.41	56.83	
	Aromatic hydrocarbons (**8**, **36**)				1.05	1.06	-	0.41	
	**Total identified**				**98.07**	**91.27**	**99.30**	**98.66**	

*R*t—retention time; RI_exp._—experimental retention index; RI_lit._—literature retention index; MS—mass spectra; tr—in traces; * *γ*-terpinene + benzeneacetaldehyde. Each analysis was performed in duplicate (*n* = 2).

**Table 4:** Antimicrobial activity of fennel essential oils from different plant parts.

Bacteria	*S. aureus*	*B. cereus*	*E. coli*	*P. aeruginosa*
Plant Part	Disc Diameter (mm)			
Stems	n.a.	n.a.	7.2 ± 0.8 c	n.a.
Leaves	n.a.	n.a.	7.7 ± 0.6 c	n.a.
Flowers	n.a.	n.a.	9.7 ± 0.6 b	n.a.
Fruits	n.a.	n.a.	14.3 ± 1.5 a	n.a.

n.a.—not active (disc diameter < 6.5 mm). Values with different letters (a–c) within the same column differ significantly (*p* < 0.05).

## Data Availability

The original contributions presented in the study are included in the article, further inquiries can be directed to the corresponding authors.

## Abbreviations

The following abbreviations are used in this manuscript:

ANOVA	analysis of variance
ATCC	American Type Culture Collection
CFU	colony forming unit
EO	essential oil
EUCAST	European Committee on Antimicrobial Susceptibility Testing
GC-MS	gas chromatography-mass spectrometry
HS-SPME	headspace solid-phase microextraction
i.d.	inner diameter
*m*/*z*	mass-to-charge ratio
MHA	Mueller-Hinton Agar
MHB	Mueller-Hinton Broth
MS	mass spectra
n.a.	not active
RI	retention index
*R*t	retention time
SPE	solid-phase extraction
st	standard compound
tr	in traces
